# “Till Death Do Us Part”: A Potential Irreversible Link Between Aberrant Cell Cycle Control and Neurodegeneration in the Adult Olfactory Bulb

**DOI:** 10.3389/fnins.2018.00144

**Published:** 2018-03-09

**Authors:** Saad Omais, Carine Jaafar, Noël Ghanem

**Affiliations:** Department of Biology, American University of Beirut, Beirut, Lebanon

**Keywords:** adult neurogenesis, olfactory bulb, cell cycle control, cell cycle proteins, tumor suppressors, aging, neurodegeneration

## Abstract

Adult neurogenesis (AN) is an ongoing developmental process that generates newborn neurons in the olfactory bulb (OB) and the hippocampus (Hi) throughout life and significantly contributes to brain plasticity. Adult neural stem and progenitor cells (aNSPCs) are relatively limited in number and fate and are spatially restricted to the subventricular zone (SVZ) and the subgranular zone (SGZ). During AN, the distinct roles played by cell cycle proteins extend beyond cell cycle control and constitute key regulatory mechanisms involved in neuronal maturation and survival. Importantly, aberrant cell cycle re-entry (CCE) in post-mitotic neurons has been strongly linked to the abnormal pathophysiology in rodent models of neurodegenerative diseases with potential implications on the etiology and progression of such diseases in humans. Here, we present an overview of AN in the SVZ-OB and olfactory epithelium (OE) in mice and humans followed by a comprehensive update of the distinct roles played by cell cycle proteins including major tumors suppressor genes in various steps during neurogenesis. We also discuss accumulating evidence underlining a strong link between abnormal cell cycle control, olfactory dysfunction and neurodegeneration in the adult and aging brain. We emphasize that: (1) CCE in post-mitotic neurons due to loss of cell cycle suppression and/or age-related insults as well as DNA damage can anticipate the development of neurodegenerative lesions and protein aggregates, (2) the age-related decline in SVZ and OE neurogenesis is associated with compensatory pro-survival mechanisms in the aging OB which are interestingly similar to those detected in Alzheimer's disease and Parkinson's disease in humans, and (3) the OB represents a well suitable model to study the early manifestation of age-related defects that may eventually progress into the formation of neurodegenerative lesions and, possibly, spread to the rest of the brain. Such findings may provide a novel approach to the modeling of neurodegenerative diseases in humans from early detection to progression and treatment as well.

## Introduction to adult neurogenesis in mammals

### Overview of adult neurogenesis as a lifetime developmental process

Neurogenesis or “the birth of neurons” is the developmental process by which mature and functional neurons are generated in the brain from a pool of neural stem and progenitor cells (NSPCs). Although neurogenesis is primarily an embryonic process, the recent discovery that it persists throughout life in vertebrates including mammals (with distinct spatiotemporal variations and cell fate restrictions across species) forced a re-assessment of the adult brain's plasticity and regenerative capacity (Bonfanti and Peretto, 2011). It is now well established that adult neurogenesis (AN) is restricted to two neurogenic sites in the adult mammalian brain: the sub-granular zone (SGZ) of the hippocampus (Hi) and the sub-ventricular zone (SVZ) lining the lateral ventricles (LV) (Ming and Song, 2011). Rather than producing massive numbers of the different neuronal subtypes as is the case during development, AN provides a continuous but limited supply of specific subtypes of young neurons that allow for qualitative contribution to existing networks in the form of structural plasticity. In addition, unlike during embryonic neurogenesis, AN is precisely modulated by the level and forms of interaction with the environment and hence is largely activity-dependent. In humans, neurogenesis is ongoing throughout life in the adult hippocampus where it is associated with the formation of new memories and learning but ceases early in the olfactory bulb (OB) during the second year of infancy.

Massive investigation is under way trying to determine whether neurogenesis may potentially carry a regenerative or possibly a restorative role inside the adult brain besides its modulatory contribution to normal brain function. So far, studies have demonstrated that the radial glia-like NSCs population found in the SVZ is regionally specified and heterogeneous and is therefore capable of generating oligodendrocytes and astrocytes (albeit at low numbers) in addition to neurons *in vivo* (Ahn and Joyner, 2005; Menn et al., 2006; Codega et al., 2014; Mich et al., 2014) and *in vitro* (Ortega et al., 2013). Moreover, consistent with the embryonic origin of adult NSPCs (Fuentealba et al., 2015; Furutachi et al., 2015), many of the cellular and molecular mechanisms controlling adult neurogenesis are notably similar to those acting during development but often display contextual differences (for review; Lim and Alvarez-Buylla, 2016). Interestingly, studies have also shown that neurogenesis is stimulated or can be affected by brain injury and various brain pathologies e.g., psychiatric disorders as well as neurodegenerative diseases such as Alzheimer's disease and Parkinson's Disease (Winner and Winkler, 2015). Nonetheless, the nature of such interactions e.g., whether direct or indirect and/or based on cause-and-effect relationship or not, is still under investigation.

### Adult neurogenesis in the SVZ-OB in mice: cell types, key regulators and function

Adult neural stem cells (aNSCs) found in the SVZ are type B1 radial glia-like quiescent cells that express a number of glial markers including Glial Fibrillary Acidic Protein (GFAP), Glutamate-Aspartate Transporter (GLAST), and Brain Lipid-Binding Peptide (BLBP). They also display regional specification whereby distinct NSCs located in different compartments along the walls of the LV generate distinct subtypes of OB interneurons (Merkle et al., 2007, 2014). Once activated, type B1 cells express Nestin and give rise to transient-amplifying cells or type C, which generate neuroblasts or Type A that migrate to the OB where they differentiate into distinct subtypes of interneurons occupying the granule cell layer (GL; ~95% of the total newborn neurons) and the periglomerular layer (PGL; ~5%) (Codega et al., 2014; Bonaguidi et al., 2016). Many signaling molecules including Shh, BMP, Wnt, Notch, and, transcription factors such as *Sox2, Ascl1, Dlx2, Pax6, Tbr2, Prox1* as well as mitogens and growth factors e.g., FGF2, EGF are common regulators of both embryonic and adult neurogenesis and act in a developmentally similar context. Yet, significant differences exist about how these factors control NSPCs properties such as cell fate determination and maintenance at the molecular level (Urban and Guillemot, 2014; Gotz et al., 2016; Lim and Alvarez-Buylla, 2016). Notably, aNSCs have much longer cell-cycle length compared to their embryonic counterparts, possibly to avoid accumulation of genetic mutations and DNA damage, premature shortening of telomeres and/or pool exhaustion (Gotz et al., 2016).

From a functional perspective, addition of newborn neurons during AN is considered a dynamic form of neuronal plasticity allowing the brain to refine its structural organization and circuitry functions in response to constantly changing interactions with the environment. At the network level, this activity-driven plasticity translates into dynamic cellular changes that are primarily occurring at the synaptic contacts such as addition of new synapses, removal of existing ones and/or relocation of others which interestingly seems to allow a faster adaptation to environmental stimuli as it has been recently suggested (Hardy and Saghatelyan, 2017). As a result, a tight regulation of AN is required to maintain the proper balance between circuit stability and plasticity (associated with the addition of new neurons) in order to preserve normal brain function. In fact, while the absolute number of newborn neurons depends on the rate of proliferation in the SVZ, about 40–50% of these neurons are turned over in the rodent OB primarily by apoptosis while the rest survive for less than 18 months (Winner et al., 2002; Whitman and Greer, 2009). Importantly, this turnover mechanism by apoptotic death peaks during a “critical period” between 14 d and 28 d after cell birth (Yamaguchi and Mori, 2005). It is also a highly dynamic process that is largely influenced by environmental stimuli and sensory input such as experience-induced alterations or challenging associative sensory tasks. For instance, studies have shown that sensory deprivation by nostril occlusion following birth reduces the number of granule cells by increased apoptosis whereas exercise or olfactory enrichment triggers the opposite effect (Corotto et al., 1994; Petreanu and Alvarez-Buylla, 2002; Rochefort et al., 2002).

Two central questions are still the subject of extensive research concerning the contribution of AN to the OB circuitry: (1) is adult neurogenesis necessary for normal olfactory function or specific olfactory tasks only? (2) What are the cellular/molecular properties that distinguish newborn adult neurons from perinatal mature neurons and thus render their influence on circuit function unique? Using variable genetic and chemical approaches to disrupt AN, several studies have attempted to answer these questions and showed that AN is likely dispensable for normal odor and threshold detection but can still accelerate discrimination learning e.g., during operant associative olfactory tasks and enhance long-term memory (reviewed in Lazarini and Lledo, 2011; Alonso et al., 2012). Moreover, adult-born neurons seem to differ from mature GCs born around birth by their degree of excitability and plasticity as well as their targets. This is directly linked to their synaptic properties including synaptic maturation and output, such as a resistance to synaptic depression mediated by the metabotropic GABAb receptor as well as synaptic refinement through re-location (Breton-Provencher and Saghatelyan, 2012; Valley et al., 2013; Hardy and Saghatelyan, 2017).

### Adult neurogenesis in the SVZ-OB in humans compared to rodents

Adult neurogenesis in the OB is not restricted to rodents and has been described in other mammalian species including adult monkeys (Kornack and Rakic, 2001). But when compared to other mammals including primates, the adult human SVZ is occupied by a “ribbon” of GFAP-expressing astrocytes lining the ependymal layer, thus lacking the classical cellular organization observed in rodents and described above (types B, C, and A cells) (Sanai et al., 2004). Early studies have reported the presence of ongoing neuroblast migration between the LV and OB and the generation of newborn neurons in the adult human OB (Bedard and Parent, 2004). However, later reports argued for the lack of substantial neurogenic activity in the SVZ and the absence of high number of dividing neuroblasts along the RMS in the adult human brain. Instead, they revealed high proliferation only in the human fetal brain with cells co-expressing the migrating neuroblast markers Double-Cortin (DCX) and Polysialylated Neural Cell Adhesion Molecule PSA-NCAM (Sanai et al., 2011; Wang et al., 2011). In addition, Sanai et al. identified a second migratory stream of DCX+ cells heading to the ventromedial prefrontal cortex in the fetal brain and noted that the rate of proliferative and migratory activities in the SVZ and RMS respectively is greatly reduced after 8 months of age (Sanai et al., 2011). More recently, robust SVZ migration was further extended to the whole infant frontal lobe (up till 5 months of age), specifically the anterior cingulate cortex (Paredes et al., 2016a). Also, in favor of the lack of bulbar neurogenesis in humans is a ^14^C birthdating analysis of OB neuronal DNA showing around 0.008% neuronal turnover in the human OB annually (less than 1% of neurons being replaced per 100 years) (Bergmann et al., 2012) compared to about 50% in rodents (Imayoshi et al., 2008). The discrepancy observed among the above studies could partially be due to the existence of resident NSCs in the adult human OB (Pagano et al., 2000). In terms of relative volume, the OB makes up around 0.01 and 2% of the human and mouse brain, respectively (McGann, 2017), which can also be accounted for by the negligible addition of newborn OB neurons in humans (Ernst and Frisen, 2015). Some researchers have argued that the longer migratory path i.e., 50–60 mm-long adult human RMS compared to 30 mm in the fetal brain on average and, the structural complexity of the adult brain reflect unique challenges facing immature neurons in order to successfully reach the adult OB (Paredes et al., 2016b). Others, however, are more skeptical about the proposed correlation between a higher number of OB neurons and more functional significance (Lledo and Valley, 2016), especially since the human OB has a higher glomeruli-to-olfactory receptor ratio (~16) compared to mice (~2) (Maresh et al., 2008) and is uniquely distinct of other brain regions with respect to its relative size (Finlay and Darlington, 1995). Indeed, the number of human OB neurons falls within the same order of magnitude of all mammals (around 10 million cells) and this would even explain why, contrary to a widespread misconception, human olfaction is as successful and efficient as that of other mammals (McGann, 2017).

As described above, the appearance of a hypocellular gap occupied by astrocytes but is devoid of neuronal cell bodies in the subventricular ependyma in adult humans (Quinones-Hinojosa et al., 2006) suggests a connection between loss of proliferation in this specific layer after the infancy period (around 18 months) and lack of adult bulbar neurogenesis (Paredes et al., 2016b). Interestingly, Ernst et al. established the striatum as a third site of adult neurogenesis that is unique to humans, by the detection of IdU-retaining interneurons in postmortem tissue from cancer patients treated with this analog. Using carbon dating, the same group also showed a 2.7% turnover rate in the previous subpopulation per year (Ernst et al., 2014) and later speculated that the newborn striatal neurons are SVZ-derived (Ernst and Frisen, 2015). The origin of these striatal interneurons remains uncertain though since other studies have reported different origin(s) of these cells such as the medial ganglionic eminence (Wang et al., 2014; Lepousez et al., 2015) or possibly local parenchymal astrocytes as reported after stroke in mice (Magnusson et al., 2014). Nonetheless, the implications of striatal neurogenesis having a SVZ-derived origin in psychiatric disorders have been discussed (Inta et al., 2016). In addition, a difference in SVZ-neuronal output, for example migration to the OB vs. the striatum, could account for shared functions associated with circuit plasticity in both regions such as cognitive flexibility involving the human striatum and associative operant learning in the OB (Sakamoto et al., 2014). In summary, human-specific variations associated with the SVZ neurogenic niche, especially during early postnatal neurogenesis, might explain the lack of bulbar neurogenesis with respect to other mammals.

### Adult neurogenesis in the olfactory epithelium of rodents and humans

Unlike the adult human OB, the olfactory epithelium (OE) in both rodents and humans is an active site of constitutive neurogenesis throughout life where new neurons are continuously generated in order to replace worn out ones under normal conditions or damaged cells after epithelial injury (Graziadei and Graziadei, 1979; Hinds et al., 1984; Hahn et al., 2005). The OE is a pseudo-stratified epithelium lining part of the nasal cavity along its apical side as well as the basal membrane at its basal side, whereby its structure and function are vastly conserved among mammals (Lane et al., 2002; Nibu, 2002). The OE contains a single type of bipolar neurons, the olfactory sensory neurons (OSNs), which extend their dendrites expressing specific odorant receptors (OR) into the nasal cavity for odor detection. OSNs relay sensory signals to the OB by projecting their axons along the olfactory nerve layer (ONL) that synapse with dendrites of second-order neurons, the mitral and tufted cells (M/T cells), inside glomeruli in the periglomerular layer (PGL) (Buck, 2004). Two cell types, the globose basal cells (GBC) and the horizontal basal cells (HBC), reside in the basal layer of the OE and are responsible for retaining lifetime neurogenesis and regeneration in rodents and also in humans, albeit at much lower rate (Schwob et al., 2017). In mice, these two populations are clearly distinguished based on their distinct morphology and molecular content (Calof et al., 1998; Mackay-Sim, 2010). However, these differences are less clearly defined and require further investigation in humans (Chen et al., 2014). The GBCs are a multipotent and actively proliferating population of stem/progenitor cells capable of generating all cell types in the developing OE and carry the day-to-day replenishment of neurons in normal ongoing turnover and post-injury in the adult tissue. They are a heterogeneous population at the molecular level, marked by the expression of different types of transcription factors at distinct stages of development including common key regulators found in other neurogenic sites e.g., *Sox2, Pax6, Ascl1, NeuroD1* (reviewed in Schwob et al., 2017). In comparison, HBCs arise later in development and are suggested to exhibit multipotent capacity. However, they are primarily quiescent under homeostatic conditions and appear to contribute to epithelial reconstitution in response to severe lesions only e.g., death of sustentacular cells. Despite the ongoing constitutive OE neurogenesis in humans, the regenerative capacity of the OE declines with age due to a decrease in number and function of stem/progenitor cells (pool exhaustion) as described below (Doty and Kamath, 2014).

## Role of cell cycle proteins during adult neurogenesis in the SVZ-OB and OE

Under normal physiological conditions, AN is controlled by an array of cell-intrinsic and extrinsic factors that exhibit complex interactions inside the neurogenic niches and regulate stem/progenitor self-renewal, proliferation and differentiation. In the SVZ, such regulatory factors include mitogens, growth factors, transcription factors, chromatin modifiers and non-coding RNAs (for review; Lim and Alvarez-Buylla, 2016). In this context, the classical cell cycle machinery comprised of tumor suppressor genes, cyclins, cyclin–dependent kinases (Cdk) and cyclin-dependent kinase inhibitors (Cdki), is the master regulator of the proliferative properties of embryonic NSPCs including quiescence (G0), self-renewal/maintenance, cell cycle length and checkpoints (G1-S, G2-M) as well as spatiotemporal regulation of cell division. Besides cell cycle control, many cell cycle proteins were notably shown to carry out “*second careers*” implicating them in the regulation of progenitor specification, neuronal commitment, neuronal migration or terminal differentiation during development (Herrup, 2013). In addition, studies have uncovered conserved but also divergent functions attributed to cell cycle proteins during AN. Interestingly, only some of these proteins turn out to be involved in cell proliferation control in the adult neurogenic sites by displaying cell-type specific functions e.g., regulation of stem cell vs. progenitor cell proliferation or tissue-specific functions inside the SVZ compared with the SGZ. In some cases, they are differentially required at distinct developmental stages e.g., young adult vs. adult vs. aged adult (for in depth reviews on cell proliferation control in the embryonic and adult brain, see Beukelaers et al., 2012; Bartesaghi and Salomoni, 2013; Cheffer et al., 2013).

**Figure 1:**
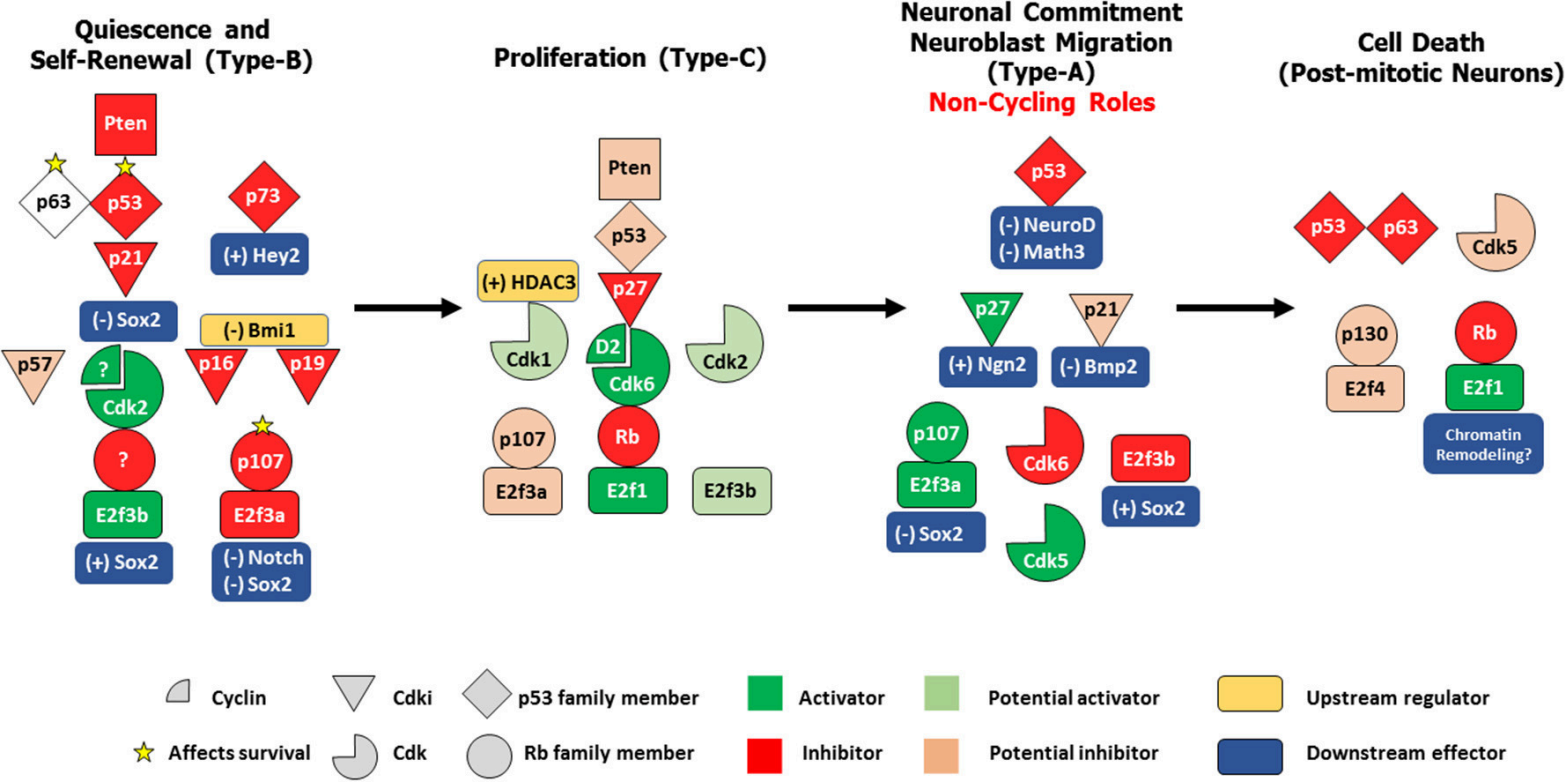
Differential control of distinct stages in adult SVZ-OB neurogenesis by different cell cycle regulatory proteins. In response to the proper cues, quiescent type-B stem cells get activated to produce rapidly dividing type-C progenitors, which in turn preferentially commit to a neuronal lineage as neuroblasts that migrates along the RMS and differentiate into post-mitotic interneurons in the OB. These four populations are under the tight control of the cell cycle machinery. In both NSCs/type-B cells and NPCs/type-C cells, a classical/general signaling pathway can be constructed based on existing literature: Pten -> p53 -> Cdki -> Cdk/Cyclin -> Rb family member/E2f. However, distinct members of these protein families seem to play non-redundant roles during these two stages e.g., p21, Cdk2, and p107 act specifically in type-B maintenance while p27, Cdk6, and Rb preferentially regulate type-C proliferation. Interestingly, within the same population, family members can have non-overlapping functions e.g., p63 and p73 in controlling survival of type B cells, or even more, might have opposing roles e.g., E2f3a and E2f3b in regulating Sox2-dependent expansion of the NSCs pool. In neuroblasts, several cell-cycle proteins switch to non-cycling tasks as they act to maintain neuronal commitment e.g., p21 and Cdk6 and neuroblast migration e.g., Cdk5. Finally, following neuronal maturation, specific regulators e.g., Rb, p53, Cdk5 act to preserve cell cycle suppression by inhibiting cell-cycle re-entry (CCE), a process prone to deregulation in the aging OB, thus potentially leading to neurodegeneration. (+); positive control, (–); negative control.

As during development, cell cycle proteins fulfill distinct non-cycling functions in the adult brain ranging from stem cell quiescence and senescence to progenitor commitment in the SVZ as well as neuroblast migration in the RMS and long-term survival of post-mitotic adult born neurons in the OB. Here, we expand and update these findings in the light of the latest data by re-constructing the distinct roles played by the cell cycle machinery according to the developmental context of each cell type found in the SVZ-OB (Figure 1). It is imperative to mention that most studies on cell cycle regulators relied on the use of germline knock-out (KO) or conditional KO mouse models which may not necessarily discriminate between primary vs. secondary effects as a result of gene deletion on adult neurogenic processes. For example, phenotypic consequences could result from developmental and/or early-postnatal alterations in the VZ-SVZ rather than adult-specific functions. Therefore, the use of inducible conditional KO or transgenic reporter lines or lineage tracing techniques e.g., labeling of adult NSPCs through stereotaxic injections of recombinant viruses into the LV will guarantee a more accurate characterization of adult-specific cycling versus non-cycling functions attributed to cell cycle proteins (Dhaliwal and Lagace, 2011; Enikolopov et al., 2015).

### Control of self-renewal and quiescence in type B aNSC

Type B1 SVZ-aNSCs are radial glia-like cells that are regionally specified and capable of unlimited self-renewal throughout life. They are known to alternate between a quiescent state and an actively dividing state where they can self-renew through two modes of divisions, symmetric and asymmetric (Bonaguidi et al., 2016). Upon activation, they primarily gave rise to distinct neuronal lineages (Nestin-positive) destined to replenish the stock of adult inhibitory neurons in the OB. Using distinct lineage tracing methods, two recent studies have demonstrated that the majority of B1 cells become regionally specified early during development and as a result originate from a sub-population of embryonic NSCs between E13.5 and E15.5. They remain largely quiescent until post-natal re-activation (Fuentealba et al., 2015; Furutachi et al., 2015). Notably, the Cdki, p57, was identified as a key factor required for maintaining quiescence in this population and later on, the generation of most aNSCs. These two functions rely on its Cdk inhibitory domain, hence the regulation of the activity of the cyclin-Cdk complexes (Furutachi et al., 2015). Although a role of p57 in aNSC maintenance was not described, it is not surprising that several other Cdki and tumor suppressor genes were shown to control self-renewal and/or quiescence of aNSCs, two defining features linked to longevity and multipotentiality of these cells. Genetic studies primarily relied on the phenotypic analyses of null mutants or inducible/conditional KO animals (targeted gene deletion) *in vivo* and cell culture *in vitro* e.g., neurosphere assays.

On top of this regulatory hierarchy is Pten, an inhibitor of Akt phosphorylation by PI3K and upstream activator of p53 (Freeman et al., 2003), which negatively regulates aNSC self-renewal and subsequent progenitor proliferation without affecting neuronal production in the OB (Gregorian et al., 2009). Conditional deletion of *Pten* in a subpopulation of aNSCs leads to enhanced OB neurogenesis and olfactory function and surprisingly no sign of NSCs exhaustion in culture. Yet, it is not clear whether this is also true *in vivo*. An equivalent negative control of NSCs self-renewal is echoed with several members of the p53 family. Loss of p53 results in significant downregulation of its direct target gene, p21^Cip1^ (Meletis et al., 2006), and gives a proliferative advantage to both slow- and fast-dividing cells in the SVZ along with their rapid differentiation (Figure 1) (Gil-Perotin et al., 2006). These findings are consistent with a previous study showing that loss of p21^Cip1^ compromises aNSCs quiescence and eventually leads to NSC pool exhaustion in 16 month-old mice (Kippin et al., 2005). This p21 function was later shown to be mediated by a direct negative regulation of the expression of the pluripotency transcription factor Sox2 (Marques-Torrejon et al., 2013). The same group also revealed that BMP2, which is under the direct negative control of p21, induces premature terminal differentiation of multipotent NSCs into mature astrocytes in p21-null mice (Porlan et al., 2013). Within the p53 family, p73 maintains aNSCs self-renewal and the neurogenic capacity inside the niche independently of p53 (Gonzalez-Cano et al., 2010) and through direct transcriptional regulation of bHLH Hey2, which in turn prevents premature neuronal differentiation (Fujitani et al., 2010). Also, p73 can compensate for p63 haplo-insufficiency (in compound double heterozygotes) and divert it from inducing p53-dependent apoptosis in NSCs to trigger cellular senescence instead (Cancino et al., 2013b; Fatt et al., 2014). Furthermore, Bmi-1 was shown to promote NSC self-renewal by repressing the senescence pathways of two Cdkis, p16^Ink4a^ and p19^Arf^ (Molofsky et al., 2005). Despite this, the age-dependent increase in the expression of p16^Ink4a^ was linked to the decline in NSPCs number and function in the SVZ-OB but not the SGZ in old mice (Molofsky et al., 2006). Downstream of all the above regulators is Cdk2 which is required for proper self-renewal and proliferation in the SVZ in young mice but not at earlier/perinatal stages. This is probably due to transient compensatory mechanisms involving Rb inactivation by increased expression of Cdk4 (Figure 1) (Jablonska et al., 2007).

The Rb protein family member p107 negatively regulates the number of aNSCs in the developing and adult brain by directly inhibiting the Notch1-Hes1 pathway (Vanderluit et al., 2004), and is required for neural progenitor commitment to a neuronal fate (Vanderluit et al., 2007). A more recent report further demonstrated that p107 specifically associate with E2f3a isoform to form a transcriptional repressor complex while E2F3b recruits RNA polymerase II to create an activator complex. These two complexes aid to fine-tune the balance between neural precursor expansion and neurogenesis via direct Sox2 transcriptional regulation in the embryonic and adult brain (Figure 1) (Julian et al., 2013). Since the loss of many cell cycle regulators described above is often associated with the depletion of the aNSC pool, it would be intriguing to examine whether this could be due to reduced or lost Notch signaling which was demonstrated to be absolutely required for the maintenance of NSCs in both neurogenic sites in the adult brain (Imayoshi et al., 2010). Finally, it is worth mentioning that, unlike Nestin-positive SGZ-radial-glia like precursors, Nestin-expressing (activated) SVZ-NSCs cannot return to a quiescent state once activated, but rather undergo a fast clonal expansion (Bonaguidi et al., 2011; Calzolari et al., 2015), a fact that could also place a limitation on the long-term neurogenic potential inside the aging SVZ.

### Control of proliferation in type C transient-amplifying cells

NSPCs proliferation in the adult mammalian SVZ is subject to tight regulation by intrinsic factors such as transcription factors and epigenetic mechanisms, and extrinsic ones such as morphogens and growth factors. Equally relevant but less understood is the effect of environmental cues and activity on SVZ neurogenesis e.g., physical exercise and learning as well as that of pathological disorders e.g., epilepsy, stroke, and Alzheimer's disease (Ming and Song, 2011). Cell-cycle proteins can act to either initiate or mediate such regulatory mechanisms. Moreover, as described above, some cell-cycle regulators control cell-type or tissue-specific functions while others display broader developmental effects. For instance, p27^Kip1^ was shown to selectively control the number of transient-amplifying progenitors or type C cells at the expense of neuroblasts produced in the SVZ without affecting stem cell proliferation (Doetsch et al., 2002). In contrast, genetic deletion of cyclin D2 but not cyclin D1 completely eliminates proliferation of all neuronal precursors (hence generation of newly born neurons) in both neurogenic zones in the adult brain (Kowalczyk et al., 2004). Furthermore, both E2F1 and E2F3 promote precursor proliferation in the SVZ and may have redundant functions. Yet, it is not clear whether this function affects NSCs and/or NPCs proliferation (Cooper-Kuhn et al., 2002). More recently, we have shown that Rb, the master regulator of the G1-S phase checkpoint, specifically regulates progenitor proliferation (but not stem cells) in the SVZ (Naser et al., 2016) and proliferation of late-born progenitors/immature neuroblasts in the SGZ (Vandenbosch et al., 2016). Hence, loss of Rb leads to enhanced prognitor proliferation in both regions. Given that both E2F1 and E2F3 are physiologically relevant Rb-interacting partners during brain development (Callaghan et al., 1999), similar interactions are likely to control progenitor proliferation during AN, although direct evidence was not established.

In return, Rb could be under the negative regulation of Cdk6, the only Cdk shown to promote NPC proliferation *in vivo* by controlling the duration of the G1 phase in committed progenitors (Beukelaers et al., 2011). Also, Rb could possibly be a downstream target of Cyclin D2 that positively regulates proliferation in the same type of cells in the SGZ (Kowalczyk et al., 2004). In comparison, HDAC3 controls progenitor proliferation by regulating the G2/M phase progression through post-translational stabilization of the G2/M kinase, Cdk1 (Figure 1) (Jiang and Hsieh, 2014). It is noteworthy that the cell cycle regulators required for maintenance, self-renewal or quiescence of aNSC population (described in the previous section) may also have an independent (direct) role in controlling NPC proliferation and not just a secondary effect. As a matter of fact, the increase in precursor expansion in E2f3a^−/−^ adult mice was directly linked to Sox2 overexpression to enhance self-renewal of aNSC at the expense of neuroblast production in both the SVZ and SGZ (Julian et al., 2013). Yet, it is still unclear whether this phenotype also reflects an additional increase in progenitor cell proliferation or not. One way to resolve this issue is to generate inducible conditional KO-reporter mice under the control of specific promoters and/or regulatory elements of NPCs specific markers such as *Mash1, Dlx2*, and *Tbr2* (Ming and Song, 2011).

### Control of lineage commitment and migration of neuroblasts

Although AN in the SVZ can produce a small population of non-myelinating and myelinating oligodendrocytes (Menn et al., 2006) in addition to astroglia in the RMS and the corpus callosum (Sohn et al., 2015), most Type-C cells end up committing to a neuronal fate where they give rise to neuroblasts that migrate along the RMS en route to the OB and differentiate into subtypes of inhibitory neurons (Whitman et al., 2009). It is at these stages of neuronal commitment and migration that some cell cycle regulators exhibit their second *careers*. Beukelaers et al. showed for instance that Cdk6 is needed for the switch between cell proliferation and neuronal differentiation in adult progenitors and its deficiency prevents this transition by lengthening the G1 phase duration (Beukelaers et al., 2011). Moreover, Gil-Perotin et al showed that p53 and p27^Kip1^ play antagonistic effects on neuroblast production in the SVZ even though both genes negatively regulate precursor proliferation (but not synergistically) as described above. In specific, p53 suppresses neuroblast generation by the negative control of the pro-neural genes NeuroD and Math3 whereas p27^Kip1^ promotes neuronal commitment by stabilizing the neurogenic transcription factor Ngn2 (Gil-Perotin et al., 2011). Again, the level of Sox2 expression critically affects the final output of neurogenesis whereby high Sox2 levels mediated by E2F3b trigger precursor proliferation at the expense of neuronal differentiation while low Sox2 levels as induced by E2f3a/p107 lead to the opposite effect (Julian et al., 2013). Importantly, Cdk5, a neuronal protein kinase and non-conventional cell cycle protein (does not bind to cyclins), is required for proper neuroblast migration in the adult RMS. Its loss hence impairs the speed, directionality and leading process extension of neuroblasts in a cell-autonomous fashion (Figure 1) (Hirota et al., 2007).

### Control of terminal differentiation and survival of adult-born neurons in the OB

It is estimated that 20,000–30,000 neuroblasts arrive at the rodent OB every day and, 4 weeks later, 50% of these immature adult-born granule cells are eliminated by natural turnover. The remaining interneurons survive up to 12 months in mice and 19 months in rats before getting turned over by another round of newborn neuroblasts (Breton-Provencher and Saghatelyan, 2012). Of note, it was believed for a long time that neurons are permanently post-mitotic and as a default state, they cannot re-enter the cell cycle. Recent evidence has strongly challenged this dogma and studies have shown that neuronal cell-cycle re-entry (CCE) could be triggered by many assaults e.g., accumulation of DNA damage with aging and oxidative stress (see next section). Thereafter, several cell-cycle proteins are crucially required to keep neurons in an arrested state at the G0 phase (cell cycle suppression) by carrying “non-cycling” functions (Herrup and Yang, 2007). The OB is no exception as we have recently shown a critical requirement for Rb in the long-term survival of adult-born OB neurons (1–4 month old). This is despite the fact that loss of Rb does not have any obvious effect on terminal neuronal differentiation or integration into existing networks (Naser et al., 2016) as seen during embryonic brain development (Ghanem et al., 2012).

In this context, Rb might act, as in NPC proliferation, to repress the function of classical cell cycle genes such as E2f1 and/or E2F3 as well as E2F-induced apoptotic genes (Cooper-Kuhn et al., 2002). However, many observations argue against this hypothesis. Instead, the resulting cell death due to loss of Rb may not depend on classical E2f-regulated apoptotic targets such as Puma and Apaf1 but rather on other mechanisms. In fact, induced deletion of Rb in post-mitotic cortical neurons was shown to trigger markers of DNA damage and DNA repair enzymes at a time the classical p53 and E2f-mediated responses do not appear to be initially activated (Andrusiak et al., 2012). Also, the Rb LXCXE binding domain is dispensible for cortical cell death, which instead is caused by E2f-responsive chromatin modeling (Andrusiak et al., 2013). In line with this, generating inducible conditional deletions of both Rb and p53 using Nestin-Cre^ERT2^-Rosa26^YFP^/double floxed mice does not rescue cell death in Rb-null adult-born neurons (Saliba A and Ghanem N, unpublished data). Moreover, loss of p53 alone also compromises long-term survival in the same neurons eventually leading to their apoptotic death through p53-independent mechanisms (Saliba A and Ghanem N, unpublished data). Consistently, Gil-perotin reported an increase in p53-independent cell death in the SVZ in p53-null mice (Gil-Perotin et al., 2006). Given the latency of cell death induced following the loss of Rb in the adult brain, it would be interesting to examine whether p130, an Rb-related family member and major regulator of neuronal survival, can compensate, at least transiently, for the absence of Rb as in the case of cultured cortical neurons where p130-E2F4 complex recruits the chromatin modifiers HDAC1 and Suv39H1 to promote gene silencing and neuron survival (Liu et al., 2005).

Cdk5 acts a potent cell cycle suppressor in post-mitotic neurons *in vivo* and in primary neurons in culture by forming a dimer with p35 and sequestering E2F1 to disrupt the E2F1-DP1 dimer (Zhang et al., 2010; Zhang and Herrup, 2011). In addition to Rb and p53, it would be interesting to determine whether Cdk5 plays a pro-survival role in adult-born OB neurons given that it is needed for maturation and survival of adult-born granule neurons in the DG where it is specifically activated by p35 (Jessberger et al., 2008; Lagace et al., 2008). p107 does not seem to play a role in survival of post-mitotic neurons although increased apoptosis was observed in the SVZ of p107-null mice. This is likely to offset the enhanced rate of stem cell self-renewal (Vanderluit et al., 2004). Alternatively, p63 protects against cell death in the OB (Figure 1) (Cancino et al., 2013b). Finally, given all the above, it should be emphasized that the dual role played by key cell cycle proteins in cell cycle suppression and neuronal survival warrant further investigation especially that CCE is fatal and often converges with neurodegeneration in many diseases (see next section).

### Cell cycle proteins during OE neurogenesis

Despite the considerable progress made in understanding the regulatory roles played by cell cycle proteins in the control of distinct phases of the cell cycle during AN in the SVZ and SGZ, cell cycle control in the developing and adult OE is still poorly investigated. p63, a p53 gene family member, was shown to be the main regulator to maintain the quiescent state of HBCs reserve pool in the OE. Following harsh OE injury, p63 expression is downregulated in order to induce the exit of HBC from quiescence and their subsequent activation to allow OE reconstitution (Schnittke et al., 2015). In a recent study, we have shown that Rb controls late progenitors' proliferation in the developing OE and the establishment of proper synaptic connections between OE-OB. Importantly, Rb is also required for terminal maturation and survival of OSNs, which is consistent with its role in proliferation control and neuronal survival in other brain regions (Jaafar et al., 2016). Whether Rb plays a similar role in the adult OE remains to be investigated.

## Cell cycle control in the adult SVZ-OB, olfactory dysfunction and neurodegeneration

### Tumor suppressor genes and neuro-oncogenesis in the SVZ-OB

Brain tumors are among the most aggressive and fatal cancers in humans and are classified into subtypes based on histologic features (Kleihues et al., 2002; Louis et al., 2007; Fuller, 2008). Gliomas are thought to originate from glial cells (due to expression of glial markers) and are divided into low-grade gliomas (LGG; type I and II) and high-grade gliomas (HGG; type III and IV) based on invasiveness and proliferation (Ghotme et al., 2017). Embryonic tumors, on the other hand, are thought to originate from neuronal progenitor cells, due to expression of neuronal markers and are classified based on their location: e.g., medulloblastoma occur in the posterior fossa and pineoblastoma arise in the pineal region. These are collectively termed “primitive neuro-ectodermal tumors” or PNETs. Type IV gliomas, primarily glioblastoma multiform, are the most aggressive brain tumors. Studies done in mice have uncovered important developmental aspects of these tumors however without reaching a consensus about their cell-of-origin. Moreover, the role of the SVZ neurogenic niche in the formation and propagation of brain tumors have been tackled and, as expected, SVZ-NSPCs transformation was correlated with loss of function of cell cycle proteins, typically tumor suppressor genes e.g., p53, Rb, Pten, and NF1. Thus, it was proposed that SVZ-NSCs/NPCs could be the initiating cells during gliomagenesis based on several shared features with tumor cells such as multipotency, expression of stem cell markers e.g., Sox2 and Nestin, and, responsiveness to extrinsic signals e.g., Sonic Hedgehog (Kusne and Sanai, 2015). For instance, following the exposure of p53^−/−^ mice to the mutagen *n*-ethyl-*n*-nitrosourea (ENU), glioblastoma-like tumors can form in periventricular locations and are characterized by enhanced NSCs self-renewal, recruitment to the fast-proliferating progenitor population (type C) and impaired differentiation (Gil-Perotin et al., 2006). Consistently, other studies showed that conditional deletions of p53 in combination with NF1 (Neurofibromin I) and/or Pten in adult astrocytes or NSPCs primarily resulted in the formation of HGGs and, less frequently, LGGs and medulloblastomas (Zhu et al., 2005; Alcantara Llaguno et al., 2009; Wang et al., 2009).

Moreover, by generating various combinations of Rb, p53 and Pten deletions, Jacques et al. reported that GFAP-expressing NSCs, but not astrocytes, gave rise to brain tumors irrespective of their location. In addition, loss of Rb was essential for developing PNET whereas Pten and p53 induce glioblastoma formation through upregulation of Cdk4 (Jacques et al., 2010). More recently, Qi et al. showed that overexpression of PIKE-A (Phosphoinositide 3-kinase enhancer) and Cdk4 in p53-Pten double KO glioblastoma mouse model synergistically shortens the latency of tumor onset and survival compared to control mice (Qi et al., 2017). In contrast with the study by Jacques et al., another study showed that combinations of p53, Rb and Pten conditional inactivation induced by GFAP-Cre^TM^ gave rise to high-grade astrocytomas (HGAs; type III) in adult mice which is consistent with concurrent mutations of these pathways in human HGAs (Chow et al., 2011). The same study argued that some of these tumors may possibly originate from mature astrocytes, while others also suggested reactive astrocytes following brain repair (de Weille, 2014), although formal evidence for both is still lacking.

Oncogenic events in the SVZ can faithfully spread to the OB, albeit reportedly infrequent. For instance, following the inducible dual KO of Rb and p53 in GFAP-Cre^TM^ mice (Chow et al., 2011), low incidence of OB tumors was described, with a “strong resemblance to human olfactory neuroblastoma” (a rare malignant neuroectodermal tumor which leads to unilateral nasal obstruction and nosebleeds, Lubojemska et al., 2016). Moreover, human glioblastoma cells injected into the striatum of immune-deficient nude mice were shown to migrate to the SVZ and subsequently to the OBs while still being potentially tumorigenic (Kroonen et al., 2011) (for a recent review on the role of cell cycle proteins in neuroblastoma cell differentiation, refer to Partridge et al., 2017). Even more rarely diagnosed are nasal gliomas in adult humans (Xie et al., 2015).

Notably, all the above studies concluded that loss of one tumor suppressor gene was not sufficient to induce tumor formation in mice, while the accumulation of cooperative mutations (or induced inactivation) in two or more tumor suppressors is required for gliomagenesis in the adult brain (still with relatively late onset). Given this and the fact that loss of many of the above genes including Pten (Gregorian et al., 2009), Rb (Naser et al., 2016), and p53 (Gil-Perotin et al., 2006; Saliba A. and Ghanem N., unpublished data) is incompatible with long-term neuronal survival in the SVZ/OB or following cortical damage (as described above), we further emphasize the hypothesis that cancer and neurodegeneration may be operating along overlapping/convergent pathways at least partially. Accordingly, and depending on the cellular context, alteration of specific cell cycle genes may lead to either process e.g., tumorigenesis in proliferating cells or neurodegeneration in post-mitotic cells (Morris et al., 2010; Jabir et al., 2015). Further support to this comes at the clinical level where it was reported that tumor formation and neurodegenerative diseases seem to be mutually exclusive to a large degree. For instance, a recent meta-analysis conducted on nine published studies of AD concluded that patients diagnosed with Alzheimer's disease (AD) are 45% less likely to be at risk of cancer (Shi et al., 2015). A similar study has previously revealed a 27–38% reduced risk of cancer in Parkinson's disease (PD) patients (Bajaj et al., 2010). We therefore support the point of view that, in mature neurons including adult-born OB neurons in mammals, neurodegeneration may result from accumulation of genetic insults e.g., loss of cell cycle suppression, DNA damage, oxidative stress and/or following aging, all of which may predominantly lead to CCE (see next sections) although the potential mechanisms involved warrant further investigation.

### From olfactory dysfunction to neurodegeneration: one fatal journey implicating the OB

**Figure 2:**
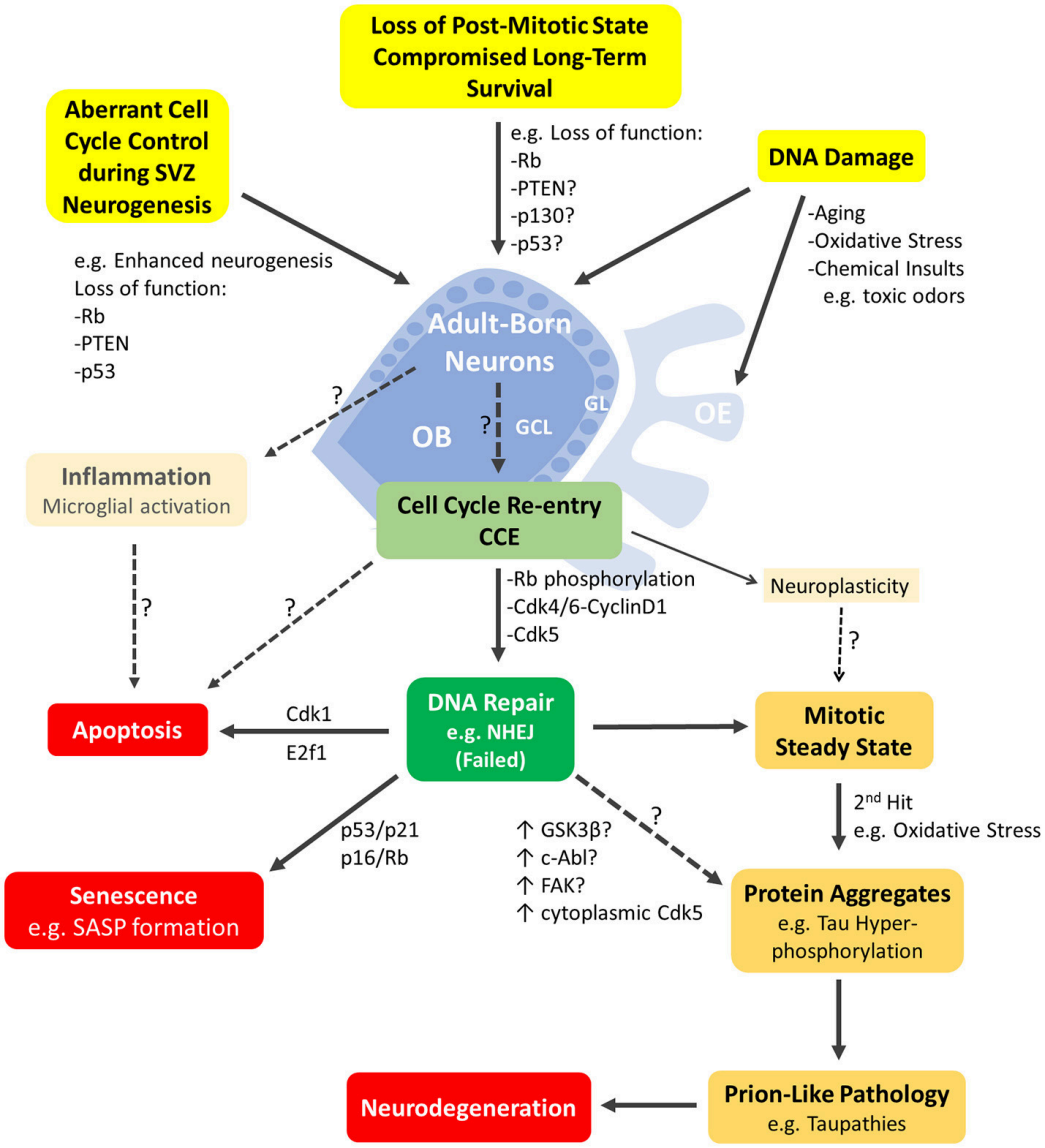
Schematic representation of the major cellular events mediated by cell-cycle proteins and likely to be underpinning neurodegeneration in the adult olfactory bulb. Adult born neurons in the olfactory bulb (OB) can re-enter the cell cycle (CCE; light green box) following various cellular events (highlighted in yellow boxes). These include DNA damage or aberrant cell cycle control due to loss of function mutations in key cell cycle genes such as Rb, p130, p53, Pten or others. CCE (G1/S phase checkpoint) requires phosphorylation of Rb by Cdk4/Cyclin D1 among others mitogenic events. CCE primarily triggers DNA repair (dark green box) but may be possibly accompanied by inflammatory response of resident microglia and/or non-mitogenic signaling such as neuroplasticity. Neurons with failed DNA repair may proceed to the S-phase and undergo: (1) apoptosis e.g., mediated by E2F1, (2) senescence induced by p53 or p16 (with manifestation of senescence-associated secretory phenotype (SASP) (red boxes) or (3) a mitotic-steady state with accumulation of additional insults that will lead to protein aggregation(s)/pathology (yellow boxes) and neurodegeneration eventually (red box). The early steps of prion-like pathologies e.g., Tau phosphorylation may be triggered by several potential kinases with upregulated activities such as cytoplasmic Cdk5, GSK3β, c-Abl and/or others. Refer to text for references. OE, Olfactory epithelium; GCL, granule cell layer; GL, glomerular layer.

At a time when cell cycle deregulation in SVZ-NSPCs is tightly linked to neuro-oncogenesis, olfactory dysfunction is strongly correlated with the initiation and progression of neurodegenerative mechanisms in the aging brain. Therefore, we propose that the OB may be a well suitable model to study age-related defects, many of which are hallmark features of neurodegenerative diseases. A growing body of evidence supports this hypothesis. First, olfactory dysfunction is a common and early symptom of major neurodegenerative diseases, namely AD, PD, Huntington's disease, amyotrophic lateral sclerosis (ALS) and others (Attems et al., 2014). In fact, it was shown that PD progression is linked to an increasing impairment of olfactory function or hyposmia which can be a useful marker of early disease development (Berendse et al., 2011) while severe hyposmia anticipates the development of PD's dementia (Baba et al., 2012). Similarly, olfactory deficit assessment might be an adequate prognostic tool to predict the conversion from mild cognitive impairment (MCI) to Alzheimer's dementia (Conti et al., 2013). Interestingly, these observations are also corroborated at the level of the underlying pathophysiology, where the OB is among the early structures to display deposits of protein inclusions such as hyperphosphorylated tau starting at Braak's stage 0 and I in AD (Kovacs et al., 2001) and α-Synuclein in PD (Braak et al., 2003). Furthermore, it was proposed that the OB could be the initiation site for the spread of these pathologies within the brain, which could occur in a prion-like manner and could hence be used to investigate the network-driven underlying mechanisms i.e., from entry of pathogens through the OE to sensitivity of the OS to oxidative stress and inflammation (Figure 2) (Rey et al., 2016).

Second, aging and/or loss of cell cycle suppression (hence post-mitotic state) can lead to CCE in mature neurons which is believed to be mechanistically related to the onset of AD and PD pathophysiology and once again, places the OB at the early stages of this process as described below. Third, since aging is the major risk factor of most neurodegenerative diseases and affects all brain regions, alterations in the aged SVZ and/or OE can trigger pro-survival compensatory responses in the OB involving mechanisms that are strikingly similar to those observed in neuronal anomalies of neurodegenerative diseases (Figure 2). We propose that future studies should aim at investigating whether cell-cycle defects affecting the maintenance of the SVZ niche and/or the OB-OE circuitry may indeed translate into manifestation of protein inclusions that are characteristic of neurodegenerative diseases, specifically AD and PD. In the next sections, we will review the evidence supporting the above observations as well as the effect of aging and neurodegeneration on AN in both the OB and the OE.

### Cell cycle re-entry in mature neurons and neurodegeneration: is it likely in the OB?

**Figure 3:**
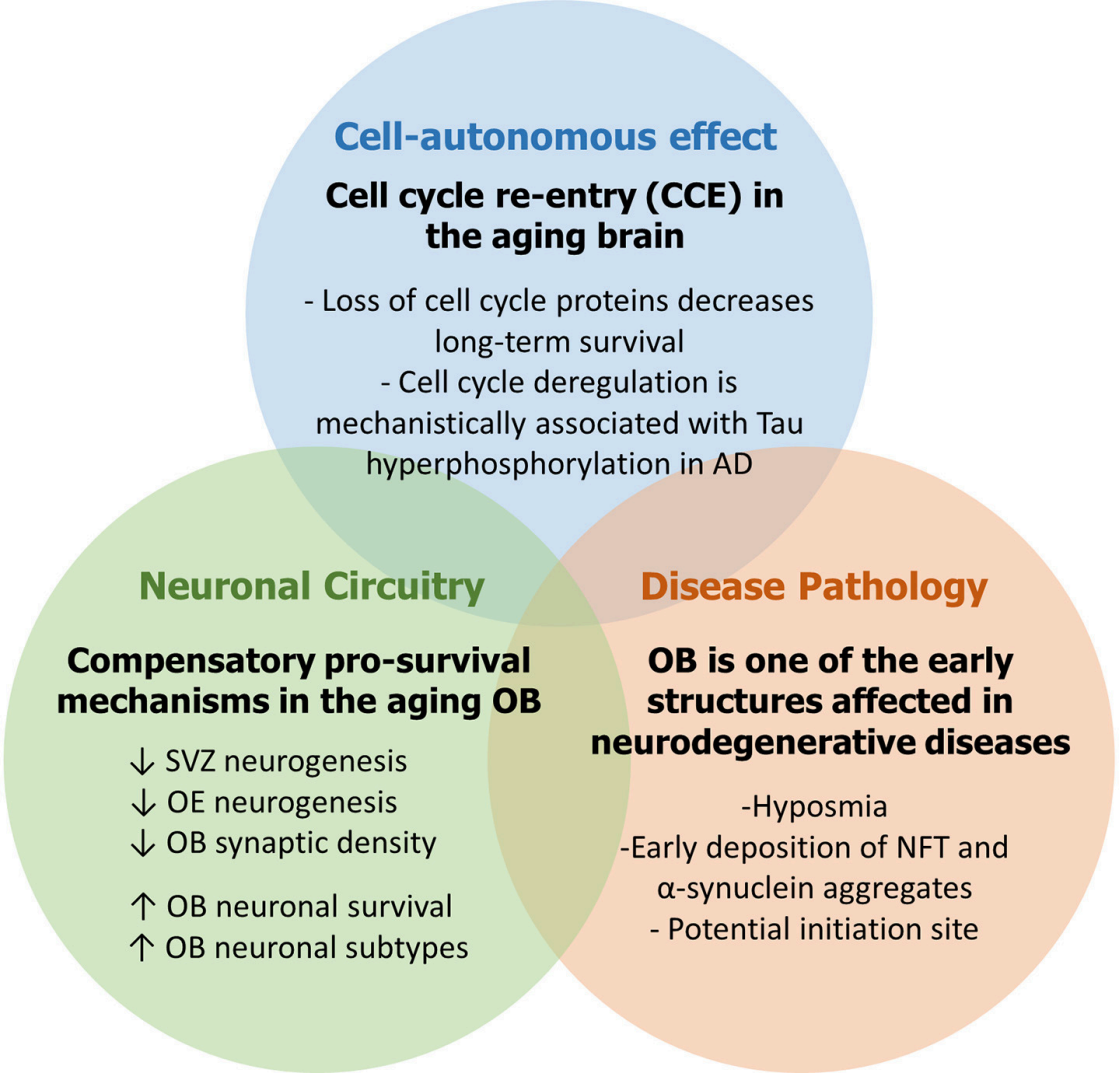
The aging OB as working model to study neurodegenerative diseases.

Several cell cycle proteins including Rb, p130 and Cdk5 among others are known to maintain a post-mitotic state in differentiated neurons, a process that is disrupted in major neurodegenerative diseases (Arendt, 2008). Loss of cell cycle suppression in mature neurons or loss of DNA integrity (which may lead to CCE) promotes an apoptotic cascade event that mimics neurodegeneration in AD and PD as some have argued (Figure 3) (Folch et al., 2012). In fact, on a more mechanistic level, neuronal CCE activates DNA repair machinery through a non-homologous end joining (NHEJ) response (Chow and Herrup, 2015). However, if DNA damage is unrepairable, some have suggested that instead of triggering cell death, the cell can commit to the senescence-associated secretory phenotype (SASP)—a mechanism by which mitotic cells evade cell-cycling especially in response to aging. Moreover, these senescent-like neurons can promote induction of the same phenotype in surrounding cells thus driving age-related diseases (Fielder et al., 2017). Besides, aneuploidy can serve as an early molecular signature of AD onset or MCI, where a chromosomal malsegregation or tetraploid state evades subsequent cell death and can lead to neuropathogenic effects such as tau phosphorylation, abnormal conformational changes or accumulation of aggregates (Arendt, 2012). Then again, another perspective proposes a “two hit hypothesis” in AD, whereby a first *hit* of mitogenic signaling dysregulation induces CCE causing a “mitotic steady state” that is not sufficient to cause cell death. However, this comes at the expense of accumulating more insults whereby a second *hit* such as of oxidative stress then triggers neuronal degeneration (Zhu et al., 2007; Aliev et al., 2014; Khan et al., 2016). Both events are necessary and sufficient to cause the disease and may be common to other neurodegenerative diseases (Figure 3). Indeed, using three different mouse models of AD, Yang et al. demonstrated that cortical neurons undergo DNA replication 6 months before developing β-amyloid plaques (known to accumulate in AD patients) or displaying activated microglia, yet without committing to cell death. This faithfully reproduces the ectopic cell cycling seen in human AD (Yang et al., 2006). However, the temporal pattern of CCE with respect to Tau pathology may differ between animal AD model(s) and AD patients as reported using the 3xTg AD mouse (Hradek et al., 2015).

Interestingly, several studies reported that loss of cell-cycle regulators so as to trigger CCE can itself be mechanistically linked to the onset of neurodegenerative lesions. For instance, inhibition of Rb in primary cortical neurons by miR-26b, a microRNA highly expressed in pathological areas in the human brain in AD, leads to aberrant CCE with increased vulnerability to tau hyperphosphorylation (another hallmark of AD pathology) and subsequent cell death mediated by pro-apoptotic downstream targets including E2F genes and cyclin E1 (Absalon et al., 2013). Notably, the same study reported nuclear export and increased activity of Cdk5, a major kinase implicated in cell cycle suppression and previously shown to contribute to tau phosphorylation and death in post-mitotic neurons after translocating to the cytoplasm (Zhang and Herrup, 2011). Similarly, in two mouse models of aging and AD, p73 haplo-insufficiency led to early appearance of phospho-tau aggregates by increased tau phosphorylation with simultaneous activation of three tau kinases GSK3β, c-Abl, and Cdk5 (Cancino et al., 2013a). However, it is not clear whether this is a direct effect or not (Figure 3). In addition, using a stochastic simulation model, Proctor and Gray predicted that, during DNA damage, increased activity and interaction between p53 and GSK3β may anticipate the accumulation of tau aggregates overtime, which in turn causes increased levels of reactive oxygen species (ROS) and more DNA damage (Proctor and Gray, 2010). Alternatively, cell cycle activation in Alzheimer's disease could be associated with neuroplasticity whereby specific cell cycle regulators can participate in the control of non-mitogenic functions such as network stability and cytoskeletal dynamics as seen in healthy neurons (for review on this topic, van Leeuwen and Hoozemans, 2015). This being said, all of the above described mechanisms following CCE are not mutually exclusive.

The implication of CCE in the formation of neurotoxic protein inclusions is yet to be established in the adult OB, but there is compelling evidence that this is likely possible. The tau kinase GSK3β (upregulated in models of AD) is abundantly expressed in the adult OB where it is essential in mediating spontaneous neural activity and odor habituation (Xu et al., 2013). Likewise, the focal adhesion kinase (FAK), thought to regulate cyclin D1 and being also involved in AD pathology (Caltagarone et al., 2007), is deregulated early in the OB of APP/PS1 mouse model prior to β-amyloid plaque formation (Lachen-Montes et al., 2016). In addition, as described earlier, loss of cell-cycle regulators in the OB leads to neuronal cell death on the long run in mice models such as Rb cKO (Naser et al., 2016), p53-null (Gil-Perotin et al., 2006) and Rb-p53 double cKO (Saliba and Ghanem, unpublished). This suggests an ability of OB neurons to better tolerate the loss of some cell cycle proteins as a *mitotic steady state* would indicate. It is noteworthy to mention that while CCE in the young adult OB may be directly linked to a neurodegenerative role, the aged OB is more adjusted to reduce neuronal loss which can reflect different roles of cell-cycle regulators during aging (Ohsawa et al., 2009). Finally, proteomic analysis of human postmortem OBs detected a specific deregulation of the DNA damage pathway in initial AD stages and that of the cell cycle in intermediate AD stages, albeit with high heterogeneity within the same AD stage (Zelaya et al., 2015).

### Similar pro-survival mechanisms are detected during aberrant OB neurogenesis and neurodegenerative diseases

The OB has been shown to adapt relatively well to aberrations affecting SVZ-derived neurogenesis and OE-based sensory input as well as age-related defects. For instance, during young adulthood, increased neuronal survival was reported in the OB following reduced SVZ proliferation in response to striatal dopaminergic denervation (Sui et al., 2012). However, this effect is likely to be only transient and will be eventually followed by a decrease in newborn OB neurons on the long run (Imayoshi et al., 2008). Nonetheless, some compensation of olfactory circuit functions can still be triggered (Breton-Provencher and Saghatelyan, 2012) e.g., retained olfactory learning following SVZ focal irradiation (Lazarini et al., 2009) and enhanced granule cell excitability following sensory deprivation by nostril closure (Saghatelyan et al., 2005). Surprisingly, despite the age-dependent decline in SVZ neurogenesis and OE deterioration (refer to next sections), the neuronal population (both projection neurons and interneurons subpopulations) remains stable in the aged OB overall, which explains the drop in adult-born neurons turnover in the OB with aging (Ohsawa et al., 2009). However, this is accompanied by a reduction of afferent synaptic input and local modulatory circuit synapses in OB glomeruli (Richard et al., 2010) as well as a deterioration of noradrenergic stimulation in the aged OB. All of these defects lead to an impairment in olfactory perceptual learning that is independent of the level of neurogenesis (Moreno et al., 2014).

Strikingly, the aged OB displays a higher caspase-9 expression (an initiator of apoptotic cell death) but not its downstream executioner active-caspase 3 that remains at normal level (Ohsawa et al., 2009). This observation may underline a unique strategy to avoid apoptosis in the aging OB through incomplete caspase signal propagation, as seen during the “abortosis” program in AD thought to promote neuronal survival in a similar manner (Raina et al., 2001). Moreover, the progressive decrease in cell death in the aging OB over time (Ohsawa et al., 2009) can lead to a buildup of some neuronal subtypes, which resonates with an increased number of dopaminergic OB neurons detected in PD, AD and frontotemporal dementia (FTD) patients (Mundinano et al., 2011). Such increase could reflect a compensatory mechanism following the early degeneration of other neurotransmitter systems and could cause the symptomatic hyposmia of the disease (Huisman et al., 2004). Finally, in the near-zero levels of neurogenesis in the adult human OB (estimated to be <1% neuronal turnover after 100 years) (Bergmann et al., 2012), it is tempting to speculate that the human OB may rely on similar compensatory mechanisms among others to maintain a constant pool of perinatal neurons throughout life.

## Aging, cell cycle control and neurodegeneration in the olfactory system

### Effect of aging and neurodegeneration on SVZ-OB neurogenesis

Aging is an inevitable process that leads to major decline in homeostasis and regenerative capacity in many organs including the brain. Age-related aberrations manifest by various structural and functional changes at the level of specific neuronal networks thus contributing to cognitive deficit. For instance, in mature neurons, age-dependent DNA damage is due to accumulating stressors such as exposure to radiation, oxidative stress, neuronal activity, telomere dysfunction or loss of selective repair mechanisms, all of which affect genomic integrity and the so-called “neuronal health.” These scars can develop independently in different subpopulations of neurons and lead to CCE or senescence-like phenotype or apoptosis as pointed earlier, but can also create a feed-forward degenerative cycle in the aging brain that is characteristic of neurodegenerative diseases (Sedelnikova et al., 2004; for recent reviews on genomic integrity and the aging brain, refer to Chow and Herrup, 2015; Fielder et al., 2017). Several studies have demonstrated that such insults do not spare the SVZ niche, hence causing major cytoarchitectural (atrophy) and proliferative changes inside the niche (Sahin and Depinho, 2010; for recent review on this topic; see Conover and Todd, 2017).

Specifically, in response to age-dependent insults or environmental changes inside the niche, SVZ-NSPCs can display various developmental aberrations such as decreased cell proliferation, reduced neuroblast number and RMS thinning, decreased expression of stage-specific markers (Luo et al., 2006; Ahlenius et al., 2009; Mobley et al., 2013), lengthening of the cell-cycle (Tropepe et al., 1997), permanent cell-cycle exit (cellular senescence) (Ahlenius et al., 2009), decreased telomerase activity (Ferron et al., 2009), increased type-B quiescence that could be rescued by intra-cerebro-ventricular infusion of growth factors such as FGF2 and HB-EGF (Jin et al., 2003; Bouab et al., 2011), as well as layer-specific loss of synaptic density inside the OB (Richard et al., 2010). As a result, the SVZ neurogenic potential sharply declines by 50–75% in the aged brain (Conover and Shook, 2011; Shook et al., 2012) despite the continuous production of few newborn neurons (Ahlenius et al., 2009; Mobley et al., 2013). In turn, this causes several behavioral deficits in fine olfactory discrimination (Enwere et al., 2004) and olfactory perceptual learning (Moreno et al., 2014) as well as short-term memory (Rey et al., 2012). Given that many of the above developmental processes are regulated, at least partially, by cell-cycle proteins in younger mice (as described earlier and Figure 1), it would be intriguing to assess the contribution of cell cycle machinery to the aging process in the SVZ-OB. While this information is still generally lacking, few studies have reported, in the aged SVZ, changes in expression of known senescence-markers (van Deursen, 2014; Zalzali et al., 2015) although their results remain controversial. For instance, Molofsky et al. associated the decrease in neuronal progenitor proliferation and subsequent neurogenesis with enhanced p16^INK4a^ expression, which may be promoting a senescence phenotype in the aging SVZ (Molofsky et al., 2006). While this finding could not be reproduced in a later study, the authors reported an increase in p27^Kip1^ levels with older age and higher expression of p19^ARF^ in SVZ-derived primary neurosphere cultures compared with embryonic ones (Ahlenius et al., 2009). Likewise, a recent report further showed that the increased (but controlled) gene dosage of Ink4/Arf and p53 attenuates the age-dependent decline in SVZ neurogenesis and significantly improves the behavioral outcomes (Carrasco-Garcia et al., 2015). An increase in SVZ cell death, typically in neuroblasts, was also detected in old- but not middle-aged mice (Luo et al., 2006).

Finally, it is noteworthy that impaired AN was described in numerous animal models of neurodegenerative diseases and only few post-mortem studies. As highlighted earlier, alterations in neurogenesis impact pathophysiological mechanisms associated with these diseases. For instance, alpha-synuclein pathology caused decreased adult OB neurogenesis in A30P mutant mouse model of PD (Marxreiter et al., 2009) as well as following overexpression of human wild type alpha-synuclein (May et al., 2012). Cheng et al. showed a robust cell-autonomous degeneration in OSNs (via Aβ-independent mechanism) and olfactory dysfunction using mouse models expressing a humanized amyloid precursor protein (hAPP) and lacking the β-site APP cleaving enzyme 1 (Cheng et al., 2011, 2013, 2016). In summary, many of the molecular regulators of AD, PD and HD also modulate AN and display different effects on NSPCs fate by regulating cell proliferation, synaptic plasticity as well as spine and axonal morphology (for recent reviews on this topic, refer to Winner and Winkler, 2015; Horgusluoglu et al., 2017). Future studies should be aimed at investigating the molecular mechanisms affected during aging in the brain, particularly those implicating cell cycle regulators with common signaling pathways in neurodegenerative diseases.

### Effect of aging and neurodegeneration on OE neurogenesis

Studies have shown age-related changes affecting the cell dynamics and regenerative capacity during OE neurogenesis that parallel those observed following aging in the SVZ-OB. Indeed, advanced age is ultimately associated with dramatic decline in OE neurogenesis overall (up to 90% decrease between 3 and 16 months in rodents, Suzukawa et al., 2011) and also with decreased olfactory sensitivity and impaired olfactory discrimination learning in rodents and primates (for recent reviews on this topic; see (Brann and Firestein, 2014; Broad, 2017). Such alterations are linked to reduced GBC proliferation (Watanabe et al., 2009), decreased number of differentiating basal cells as assessed by *Ascl1* (*Mash1*) expression, the main pro-neural gene required for OSN production (Guillemot et al., 1993), reduced EGF signaling (Enwere et al., 2004) and apoptosis (Robinson et al., 2002). In fact, Kondo et al. reported a decline in the rates of cell proliferation and cell death in the OE with increasing age (Kondo et al., 2010). Similarly, it was shown that OSN ablation following treatment with an olfacto-toxic drug triggers neurogenesis to a lesser extent in aged mice compared to young animals, suggesting an age-related decline in neuroepithelial proliferative capacity after injury (Suzukawa et al., 2011). Notably, the previous two studies detected no change in neuronal differentiation but a slower turnover rate of mature OSNs and extended OSN lifespan in the older OE compared to the younger one. The latter process is remarkably similar to the age-related compensatory mechanisms that extend survival of adult-born neurons in the aged OB (Ahlenius et al., 2009). On the other hand, with increasing age, the OSN population becomes more vulnerable to elevated levels of oxidative stress and accumulation of DNA damage, therefore rendering it more prone to neurodegeneration as might be the case inside the OB (Mattson and Magnus, 2006). In addition, telomere shortening, a common mechanism in aged tissues, was shown to impair the regenerative capacity of the OE post-lesion only by inhibiting cell cycle progression in a p21-dependent manner but without affecting OE homeostasis during mouse aging (Watabe-Rudolph et al., 2011). This is possibly justified by the compensatory regeneration that usually rescues proliferation in telomerase-deficient organ systems with low rate of cellular turnover (Brown et al., 1997; Lee et al., 1998; Rudolph et al., 1999; Kondo et al., 2010). In a related context, microarray analysis performed on senescence-accelerated mouse models (SAM) revealed dysregulated cell cycle gene expression in the OE during aging, whereby altered cell proliferation was associated with down-regulated expression of several genes involved in DNA synthesis, mitotic spindle formation and cell cycle progression such as cyclin B1 (Getchell et al., 2003).

In humans, aging also results in a “neurogenically exhausted” OE that is characterized by a gradual loss of OSNs and GBCs but not quiescent HBCs and supporting sustentacular cells. Moreover, the OE can be replaced by metaplastic respiratory epithelium (Holbrook et al., 2005, 2011; for recent review see Schwob et al., 2017). Interestingly, similar pathological changes with respect to neurogenic exhaustion were observed in rodents following bulbectomy (OB ablation) and chemical OE lesions (Kondo et al., 2009, 2010; Suzukawa et al., 2011). As described earlier, newborn OSNs extend their unmyelinated axons along the ONL and establish appropriate synaptic connections with existing neural circuits inside the OB (Cho et al., 2009). The impact of aging on this process is still poorly studied but it is likely that successful axonal targeting may be also compromised over time as seen in other tissues e.g., mouse retinal projection neurons (Samuel et al., 2011). As a matter of fact, the number of synapses is reduced in the aged OB glomeruli (Richard et al., 2010). Yet, it is not clear whether this is due to OSN loss and/or reduced axonal targeting (Mobley et al., 2014). In summary, the molecular mechanisms underlying age-induced GBCs proliferative decline and OE deterioration warrant further investigation in both animal models and humans.

While several factors contribute to age-associated decrease in olfactory function including OE damage resulting from pathogen infections, reduced expression of specific olfactory receptors and nasal engorgement (Attems et al., 2015), other functional deficits in the OE can be related to the role played by cell cycle regulators in the modulation of neurogenesis (Brann and Firestein, 2014). For instance, age-related decrease in GBC proliferation correlates with changes in gene expression of positive cell cycle regulators such as decreased expression in several cyclin-dependent kinases e.g., Cdk1, Cdk2, Cdk4 and, all D cyclins. In addition, the expressions of specific Cdkis including p27^Kip1^, p19^Ink4d^, p18^Ink4c^, and p21^Cip1^ change in a temporal pattern between young and adult stages, which may reflect changes in stem cell quiescence, cell cycle exit and neuronal differentiation with older age (Legrier et al., 2001). Yet, it is not clear whether these genetic changes are causative in nature or correspond to secondary effects associated with aging. Since aging is a common risk factor of neurodegenerative diseases, the OE, like the OB, is among the early structures to be affected by age-related alterations and disease pathology (Brann and Firestein, 2014; Mobley et al., 2014; Rey et al., 2016). A thorough examination of the molecular mechanisms involving cell cycle proteins in early stages of the neurodegenerative process and during aging is a pressing need.

## Concluding remarks

In this review, we examined the role played by the cell cycle machinery within each stage of the adult neurogenic SVZ-OB axis (as summarized in Figure 1). While this work serves as an update of previous comprehensive reviews (Beukelaers et al., 2012; Bartesaghi and Salomoni, 2013; Cheffer et al., 2013), our aim was to further implicate AN in the etiology and pathogenesis of human neurodegenerative disorders, especially in light of very few reviews that portray AN, both hippocampal and bulbar, as mediator and not simply the subject of these anomalies (Figure 2) (Gallarda and Lledo, 2012; Winner and Winkler, 2015; Hollands et al., 2016). In addition, we propose the OB as a potential candidate to model the early non-symptomatic pathophysiology of AD and PD (Figure 3) which is grounded in its remarkable susceptibility to aging at both the cellular level (as part of the whole brain's challenge to evade cycling in the post-mitotic state, Zhu et al., 2007; Chow and Herrup, 2015) and the network level (in an attempt to compensate for reduced AN in the aged SVZ and OE, Ohsawa et al., 2009; Mobley et al., 2014). More evidence in favor of this hypothesis comes from olfactory dysfunction being an effective predictor of the subsequent symptomatic features of most neurodegenerative diseases (Attems et al., 2014). Additionally, despite our focus here on cell-autonomous processes following cell cycle deregulation (Herrup and Yang, 2007) whereby cancer and neurodegeneration are thought to share common mechanisms (Morris et al., 2010), other features of the OB mark its centrality in the disease process e.g., robust pro-inflammatory responses from resident microglia following sensory de-afferentation and traumatic brain injury (Lazarini et al., 2012; Siopi et al., 2012) as well as the age-dependent decrease in synaptic density and responsiveness to noradrenergic stimulation (Richard et al., 2010; Moreno et al., 2014).

Previous studies have discussed the diagnostic implications of this proposition, such as the detection of olfactory deficits in order to anticipate the conversion from MCI to AD (Conti et al., 2013). To ameliorate olfactory function, Broad KD recently reviewed a number of therapeutic interventions ranging from behavioral to dietary and pharmaceutical interventions, all of which are believed to act partially by increasing AN (Broad, 2017). Here, we emphasized the role played by cell cycle proteins in neurodegeneration in hopes to reiterate existing treatments to a less obvious process of neurodegenerative disorders, currently known for more drastic manifestations e.g., memory loss in AD and motor dysfunction in PD. In fact, a recent review addressed the possibility of repurposing cancer drugs for the treatment of AD, several of which proved to relieve amyloid burden and tau aggregation. However, permeability to the brain blood barrier and dose-dependent efficacy pose a limitation to their successful delivery (Monacelli et al., 2017). Interestingly, the olfactory system offers alternative mechanisms of delivery by the very means of which it is most vulnerable to environmental toxic factors, that is its direct exposure to the nasal cavity (Doty, 2008). In line with this, Kovács T reviews intranasal delivery of insulin and cholinesterase inhibitors as a promising therapeutic pathway to treat or delay AD pathology (Kovács, 2013). While more research is warranted, the olfactory system serves as a gateway for disease initiation and progression, while also an early and accessible window for therapeutic intervention.

## Author contributions

SO and CJ wrote the manuscript. NG participated in writing, edited the manuscript and provided financial support.

### Conflict of interest statement

The authors declare that the research was conducted in the absence of any commercial or financial relationships that could be construed as a potential conflict of interest.
